# Identification of the length and location of the A1 pulley combining palpation technique with palm landmarks: a cadaveric study

**DOI:** 10.1038/s41598-023-49742-6

**Published:** 2023-12-20

**Authors:** Wei-xing Zhong, Jun-hua Li, Zu-jiang Chen, Wei-jie Peng, Rui-bin Gu, Chao Chen, Yi-kai Li

**Affiliations:** 1 School of Traditional Chinese Medicine, Guangdong Province, Southern Medical University, Guangzhou, 510515 China; 2 Department of Traditional Chinese Orthopedics and Traumatology, Center for Orthopaedic Surgery, Guangdong Province, The Third Affiliated Hospital of Southern Medical University, Guangzhou, 510630 China

**Keywords:** Anatomy, Musculoskeletal system

## Abstract

Through anatomical morphology, to accumulate the relevant parameters of the A1 pulley of each adult finger. A total of 100 fingers were selected, dissected layer by layer, and the A1 pulley and neurovascular of each finger were observed. Measure the length of the A1 pulley, the distance between the needle knife insertion point and the proximal edge of A1 pulley, and the nerves and blood vessels on both sides. (1) The length of A1 pulleys of each finger is 6.18 ± 0.33 mm, 6.58 ± 0.73 mm, 5.98 ± 0.67 mm, 5.36 ± 1.08 mm, 5.63 ± 1.09 mm. (2) The distances between the needle knife entry point of each finger and the volar proper nerve of the ulnar finger are 7.00 ± 1.55 mm, 8.29 ± 1.46 mm, 5.10 ± 0.25 mm, 5.30 ± 0.24 mm, 0 mm; the distances from the volar proper nerve of the radial finger are 9.08 ± 0.87 mm, 4.70 ± 1.10 mm, 7.03 ± 0.72 mm, 6.81 ± 0.22 mm, 7.81 ± 0.57 mm. (3) The distances between the needle knife entry point of each finger and the proper volar artery of the ulnar finger are 10.40 ± 0.75 mm, 8.89 ± 0.53 mm, 6.35 ± 0.44 mm, 7.26 ± 0.16 mm, 0 mm, respectively; The distances from the volar proper artery of the radial finger are 8.75 ± 1.07 mm, 6.10 ± 0.35 mm, 11.44 ± 0.41 mm, 8.19 ± 0.60 mm, 9.78 ± 0.68 mm, respectively. The landmarks of the needle entry points are located at the position corresponding to the highest point of the metacarpal heads, except the tail finger. From the needle knife entry point to distal, cut the proximal edge of the A1 pulley longitudinally along the midline until the patient can flex autonomously, and pay attention to the distance between the two sides of 3.60–11.85 mm neurovascular bundle.

## Introduction

Trigger finger (TF), also called stenosing flexor tenosynovitis, can be described as a difference in diameters of the flexor tendon and the A1 pulley due to thickening and narrowing of the tendon sheath, located at the metacarpal head^1^. TF is one of the most common diseases of the hand occurring. The lifetime prevalence of adult population is approximately 2–3%^2,3^ and up to 20% in patients with diabetes^4^, and children is 0.05–0.33%^5–7^. It can occur at different ages and is mostly related to occupation. It is more common in manual labor and middle-aged women with the dominant hand being affected more often, also seen in infants^8^. The former is related to repeated mechanical stimulation, while the latter is mostly congenital. The most common finger is the thumb^8^.

The firtst-line treatment includes nonsteroidal anti-inflammatory medications, corticosteroid injections and splinting, and also includes Traditional Chinese Medicine such as acupuncture, massage, ointment therapy, and percutaneous release and open release^1^, which are very effective and are tailored to the severity and duration of symptoms. Surgical treatment is highly successful with low complication and recurrence rates for TF. However, percutaneous blind A1 pulley release is an alternative to the open release, but its risk–benefit relationship is under debate. Among them, ultrasound-guided percutaneous blind A1 pulley release is safer and more efficient, which is a new therapeutic trend^9,10^.

In this study, morphological anatomy and stereoscopic microscopy were used to observe the parameters of A1 pulley in 10 adult upper limb specimens, and needle knife treatment was simulated. To provide anatomical basis for needle knife therapy in the treatment of stenosing flexor tenosynovitis, and to improve the accuracy, effectiveness and safety of the treatment.

## Materials and methods

The present study was performed in fresh frozen adult human cadavers that have been donated to the Department of Anatomy, Southern Medical University (SMU), Guangzhou through the Institutional body donation program following the ethical guidelines. The protocol for this research project was approved by the Biomedical Ethics committee, SMU, and informed consent was obtained from the donors. The study design involved the dissection of 20 preserved cadaver hands from the 20 adult upper extremities (Table 1). The study period was from April 2021 to December 2021. All specimens had intact hands, no deformity, no damage, and no history of surgery^11^.

**Table 1:** General characteristics of digits.

No. digits	100
Right/left	50/50
Thumb	20
Index	20
Long	20
Ring	20
Little	20

### Main reagents, instruments, and tools

**Figure 1:**
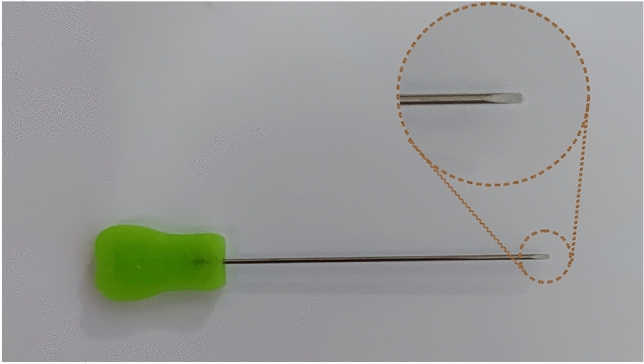
The Hanzhang needle knife. The needle knife consists of three parts: tip, body, and handle, with a 1.2 mm blade on the tip, and a 50 mm length of the body.

Pathological slicer (Shanghai Leica Instruments Co., Ltd.), stereomicroscope (Olympus SZ51-60); electronic vernier calipers (Accuracy 0.01 mm), ophthalmic scissors, ophthalmic forceps, scalpel, hemostatic forceps, skull gauge, marker, digital image acquisition (D610 camera, Nikon Corporation), image processing (photoshop 2020/Adobe Illustrator 2020, Adobe Corporation ), the needle knife ( Hanzhang Medical Instrument Co., Ltd., 1.2 × 50 mm) (Fig. 1).

#### *Step 1*

**Figure 2:**
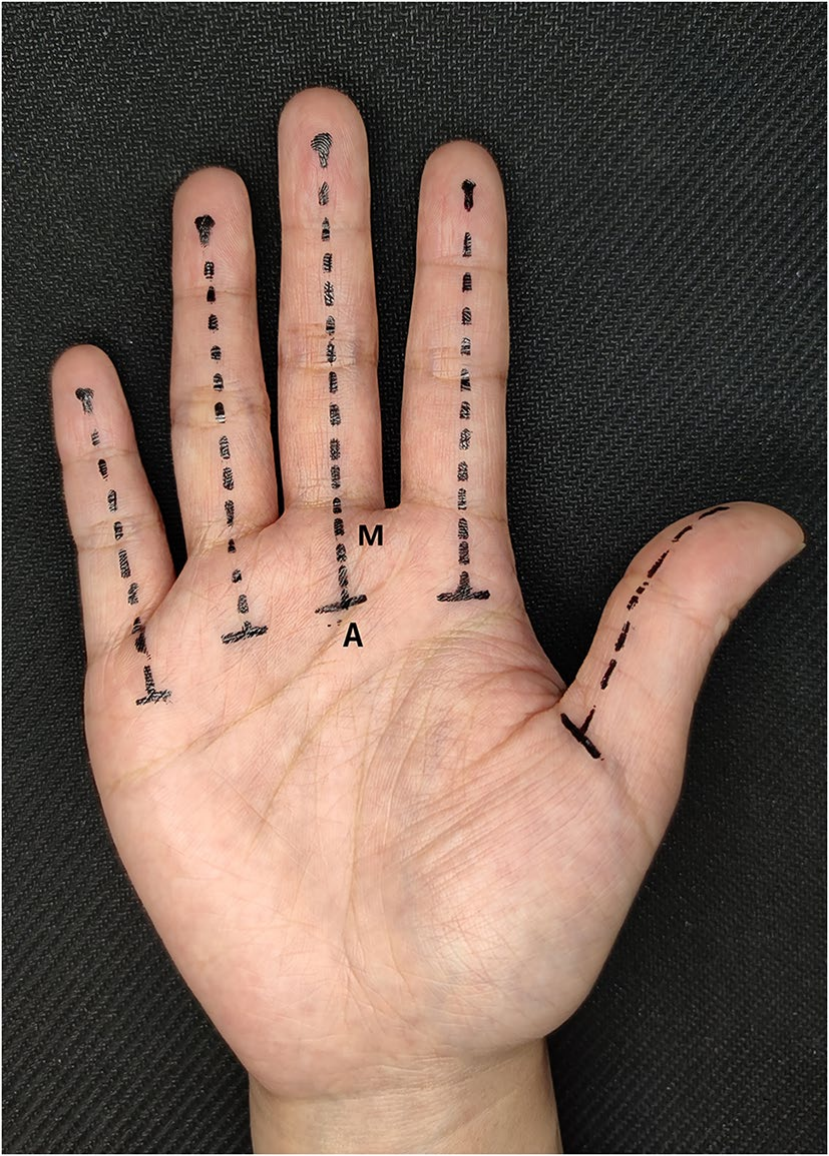
Anatomical landmarks with needle entry point.

We straighten and separate the fingers of the specimen, fix the palm face upward with needles, mark the midline (M line) of the palm face of each finger with a marker, and locate the highest point of the metacarpophalangeal joint of each finger corresponding to the palm face with a craniometer, and intersect the midline of each finger. The point is recorded as the needle entry point (A point) (Fig. 2). Use a 5 ml syringe to draw emerald green dye, and inject a little dye solution at each needlepoint. Then, the 2nd to 4th fingers were dissected in turn, the skin was incised along the midline of the fingers, the soft tissues were separated layer by layer from superficial to deep, and soft tissues such as nerves, blood vessels, flexor tendons, and tendon sheaths were exposed, photographed and recorded.

#### *Step 2*

Then, the tendon sheaths of each finger were removed. Then observed the general histological characteristics of the A1 pulley and the positional relationship between the needle insertion point and the proximal edge of the A1 pulley under a stereomicroscope.

### Description of the measurement parameters

**Figure 3:**
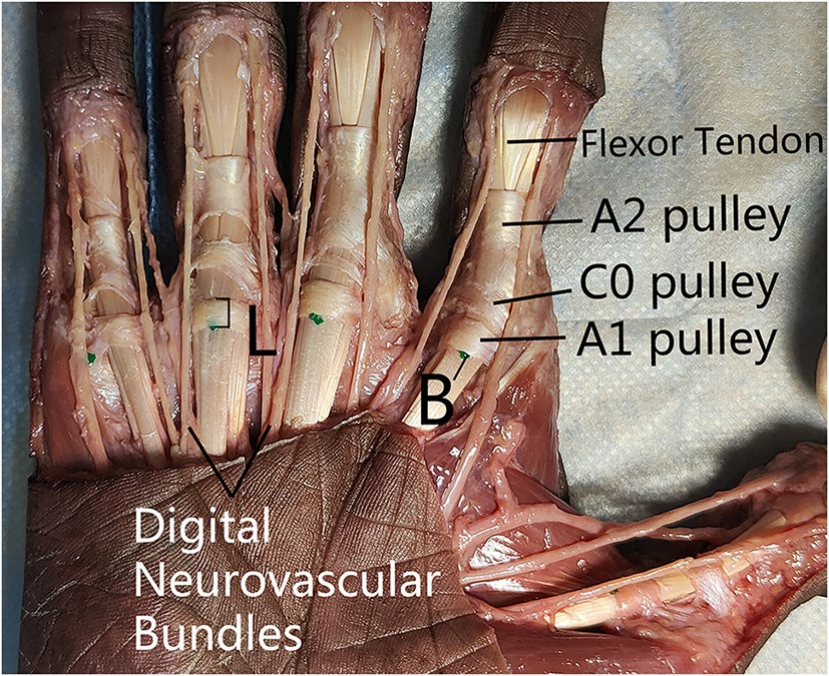
Anatomy of A1 pulley and surrounding structures. Key structures were identified and visualized: C0 pulley, A2 pulley, flexor tendon, and neurovascular bundles.

The A1 pulley, flexor tendon, and surrounding soft tissue structures were observed, and the following anatomical structures were measured using an electronic vernier caliper: the length (L) of the A1 pulley of each finger, and the distance between the midpoint of the proximal border of the A1 pulley and the volar proper nerves on both sides ( Ulnar side: N1, radial side: N2), the distance between the midpoint of the proximal border of the A1 pulley and the proper arteries on the palmar sides of the fingers on both sides (ulnar side: V1, radial side: V2), the distance (D) of the midpoint of the proximal border of the A1 pulley (B) (Fig. 3)and the needle entry point (A). The unit of distance is millimeters (mm).

### Data analysis

Depending on the type of variable, mean values, SEM values, ranges, absolute frequencies, and percentages were recorded. Analysis of variance, the Chi-square test, and the paired *t* test were used to analyze differences. A 0.05 level of statistical significance was considered significant.

### Informed consent statement

The protocol for this research project was approved by the Biomedical Ethics committee, SMU, and informed consent was obtained from the donors.

## Results

### The length of the A1 pulley

The general observation shows that the A1 pulleys of each finger are transverse fibers perpendicular to the flexor tendon, tough in texture, connected with synovial fibers at the proximal end, and difficult to distinguish, and connected with the oblique fibers at the distal end. The length of the A1 pulley of each finger is shown in Table 2. The A1 pulley of the index finger is the longest, and the A1 pulley of the ring finger is the shortest. And there was a statistically significant difference in the A1 pulley between the index finger and the tail finger (*P* = 0.015).

**Table 2:** The length of the A1 pulleys in the fingers *(*$\overline{x }$±*s,mm).*

Finger	Thumb	Index finger	Long finger	Ring finger	Little finger
L (mm)	6.18 ± 0.33	6.58 ± 0.73	5.98 ± 0.67	5.36 ± 1.08^ab1^	5.63 ± 1.09^b2^
Total F	3.252				
Total P	0.020				

a: There is a significant difference in the length of the A1 pulley between the thumb and the ring finger (*P* = 0.032, 95% CI (0.00731, 0.15689));

b1: There is a significant difference in the length of the A1 pulley between the index finger and the ring finger (*P* = 0.002, 95% CI (0.04721, 0.19679));

b2: There is a significant difference in the length of the A1 pulley between the index finger and the tail finger (*P* = 0.015, 95% CI (0.01941, 0.16899)).

### Palm landmarks of B (A1 pulley)

The distance (D) between A and B is shown in Table 3. B of the first to fourth fingers overlaps with A, while B of the little finger is (3.67 ± 0.54) mm on the radial side of A.

**Table 3:** The distance between A1 pulley and the needle entry point *(*$\overline{x }$±*s, mm).*

Finger	Thumb	Index finger	Long finger	Ring finger	Little finger
D (mm)	0	0	0	0	3.67 ± 0.54^a^

a: A is on the ulnar side of B (3.67 ± 0.54) mm.

### The distance between B and digit neurovascular bundles

Observation of anatomical specimens: two volar proper arteries of the thumb branched from the main artery of the thumb from below the A1 pulley, respectively wrapped around the inside of the sesamoid on both sides, and went to the end of the finger. Two volar proper nerves of the thumb run inside the corresponding arteries. Measure the distance between the midpoint of the proximal edge of the A1 pulley of each finger and the volar proper nerve and blood vessels on both sides of the finger, as shown in Table 4, except that B of the little finger overlaps with the ulnar digit volar proper nerve and blood vessel, the distance between the other fingers is 3.60 to 11.85 mm.

**Table 4:** The distance between B and digit neurovascular bundles *(*$\overline{x }$±*s, mm).*

Finger		Thumb	Index finger	Long finger	Ring finger	Little finger
N	N1	7.00 ± 1.55	8.29 ± 1.46	5.10 ± 0.25	5.30 ± 0.24	0
	N2	9.08 ± 0.87	4.70 ± 1.10	7.03 ± 0.72	6.81 ± 0.22	7.81 ± 0.57
V	V1	10.40 ± 0.75	8.89 ± 0.53	6.35 ± 0.44	7.26 ± 0.16	0
	V2	8.75 ± 1.07	6.10 ± 0.35	11.44 ± 0.41	8.19 ± 0.60	9.78 ± 0.68

### Observation of stereo microscope

**Figure 4:**
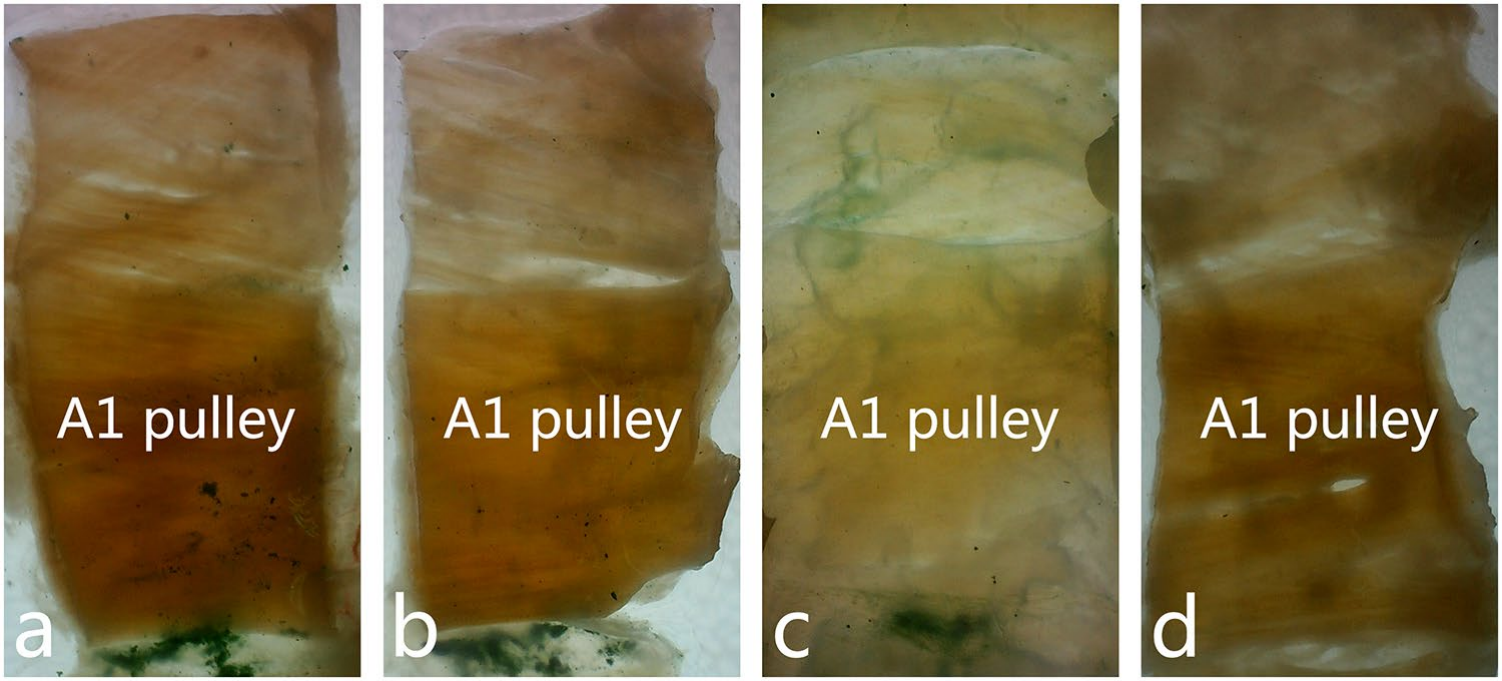
a-d are the A1 pulley of the 2nd to 5th fingers. Emerald green marked point on the proximal edge of the A1 pulley can be seen in the 2nd to 4th fingers (Original magnification ×7.8).

Observed under the stereomicroscope (Fig. 4), the A1 pulley is a dense transverse fiber with a deep yellow dense connective tissue, and both ends are continuous with the synovial fibers. The synovial fibers are thin and translucent, and are loose connective tissue. At the far end is the pale yellow C0 pulley (cruciate pulley), which is also dense connective tissue. The needle entry points (emerald green marked point) of the 1st to 4th fingers are at the proximal edge of the A1 pulley, and the needle entry point of the tail finger is at the ulnar side of the proximal edge of the A1 pulley.

## Discussion

### Histological characteristics and adjacent relationship of the A1 pulley

Observed under a stereo microscope, the A1 pulley is a pale yellow dense transverse fiber whose ends are continuous with synovial fibers and difficult to distinguish.

Literature has shown that A1 pulley is composed of an outer layer and an inner layer^12^, in which the sliding layer in the inner layer has many fibrocartilage characteristics, and the friction layer in the inner layer contains chondrocytes, which is similar to the human synovial joint and can provide conditions for the sliding of the tendon. Our research also supports this conclusion.

In addition, studies have shown that increased pressure on connective tissue leads to fibrochondrogenesis^13,14^, and specifically friction leads to cartilage transformation in the sliding layer^15^. This suggests that the pathogenesis of TF may be due to the fibrochondrogenesis of A1 pulley caused by repeated friction, which leads to the difference in diameter of tendon sheath and flexor tendon, especially the stenosis of the proximal end of A1 pulley. Therefore, in clinical practice, TF is often cured by cutting the proximal end of A1 pulley, rather than all A1 pulley. Literature has shown that the success of the operation mainly depends on the complete or incomplete release of the proximal part of the A1 pulley, while the distal part of the A1 pulley is not necessarily related to the clinical outcome^15^.

### Needle feeding position and depth

The success or failure of the percutaneous release of the TF depends on how to locate the starting point of the A1 pulley. In addition, the correct needle entry position can effectively avoid damage to the surrounding neurovascular bundles. However, we have found many papers lack precise parameters for the surface location of the A1 pulley^16,17^. Some kinds of literature suggest that the half distance (1/2 ab) between the transverse lines of the palmar fingers (a) and the transverse lines of the proximal interphalangeal joints (b), the needle entry position is at the proximal 1/2 ab of the transverse lines of the palmar fingers^18–20^. We choose the palm position corresponding to the highest point of the metacarpal head of each finger as the needle insertion point, in which the horizontal line of the metacarpophalangeal and the highest point of the metacarpal head of the thumb are at the same position, so the needle insertion point of the thumb is the intersection of the horizontal line of the metacarpophalangeal and the midline of the finger^21^. Our research shows (Table 2) that the A and B of the 1st to 4th fingers are the same, and the needle insertion point of the little finger is at the ulnar side of B (3.67 ± 0.54) mm. And research shows (Table 3), except that B of the little finger, overlaps with the ulnar digit volar proper nerve and blood vessel, the distance between the other fingers is 3.60 to 11.85 mm. The most dangerous place is the neurovascular bundle on the radial side of the index finger, and the closest distance is 3.60 mm. Strictly follow the needle insertion position and generally do not damage the neurovascular bundles on both sides^22^. In addition, for the depth of needle insertion, we suggest that the needle should be stopped after the needle tip touches the leather-like fibrosis, and the proximal edge of the A1 pulley can be cut. If it is too deep, the bursa and tendon will be cut, which theoretically will lead to tendon adhesion, which may increase the chance of recurrence, and straightening will lead to tendon rupture^17^.

### Comparison of other treatments

A clinical study showed that in the short term, the percentage of pain and scarring was higher in open surgery than percutaneous release (*P* < 0.05), and the satisfaction was significantly worse (*P* < 0.05). Long-term, however, there were no significant differences in recurrence rates, pain, scarring, and satisfaction^23^. The literature suggests that the success rate of open release ranges from 60 to 97%^24,25^. The complication rate for open release, including digital nerve injury, infection, stiffness, weakness, scar tenderness, and bowstringing of the flexor tendons, ranges from 7 to 28%. The reported rate of infection and digital nerve damage is as high as 12%^26–28^. The success rate of percutaneous release is over 90%. Complications are rare but include digital nerve injury, bowstringing (if release extends into the A2 pulley), infection, hematoma, persistent pain, and flexor tendon injury^17,29,30^.

In addition, percutaneous release combined with splint immobilization can accelerate the recovery of interphalangeal joint flexion contracture^15,31^. And ultrasound can be used as a tool for better success by means of assisting the placement of the needle during percutaneous procedures. Therefore, needle knife percutaneous release under the guidance of ultrasound visualization may be the trend of the future^10,32^.

Steroid injection therapy is more effective for early TF. Notably, the literature showed that steroids injected into the subcutaneous tissue around the A1 pulley gave better clinical outcomes than that injected into the sheath alone^33^.

The major limitation of the present study is that cadavers might have altered landmarks and tissue turgor owing to soft tissue shrinkage or fluid shifts postmortem. Also, the presence of a nodule over the A1 pulley, a thickened pulley, or a history of triggering was not a requisite inclusion in this study. Further study is required to determine the usefulness of percutaneous release in the thumb.

## Data Availability

The datasets used and/or analysed during the current study available from the corresponding author on reasonable request.
